# Critical role of microbiota within cecal crypts on the regenerative capacity of the intestinal epithelium following surgical stress

**DOI:** 10.1152/ajpgi.00294.2016

**Published:** 2016-12-15

**Authors:** Alexander Zaborin, Monika Krezalek, Sanjiv Hyoju, Jennifer R. Defazio, Namrata Setia, Natalia Belogortseva, Vytautas P. Bindokas, Qiti Guo, Olga Zaborina, John C. Alverdy

**Affiliations:** ^1^Department of Surgery, University of Chicago, Chicago, Illinois;; ^2^Pathology, Pritzker School of Medicine, University of Chicago, Chicago, Illinois;; ^3^Integrated Light Microscopy Core Facility, University of Chicago, Chicago, Illinois; and; ^4^The James Franck Institute, University of Chicago, Chicago, Illinois

**Keywords:** surgical stress, cecal crypt microbiota, Lgr5, proliferative zone, apoptosis, epithelial regeneration, fecal microbial transplant

## Abstract

Cecal crypts represent a unique niche that are normally occupied by the commensal microbiota. Due to their density and close proximity to stem cells, microbiota within cecal crypts may modulate epithelial regeneration. Here we demonstrate that surgical stress, a process that invariably involves a short period of starvation, antibiotic exposure, and tissue injury, results in cecal crypt evacuation of their microbiota. Crypts devoid of their microbiota display pathophysiological features characterized by abnormal stem cell activation as judged by leucine-rich repeat-containing G protein-coupled receptor 5 (Lgr5) staining, expansion of the proliferative zone toward the tips of the crypts, and an increase in apoptosis. In addition, crypts devoid of their microbiota display loss of their regenerative capacity as assessed by their ability to form organoids ex vivo. When a four-member human pathogen community isolated from the stool of a critically ill patient is introduced into the cecum of mice with empty crypts, crypts become occupied by the pathogens and further disruption of crypt homeostasis is observed. Fecal microbiota transplantation restores the cecal crypts’ microbiota, normalizes homeostasis within crypts, and reestablishes crypt regenerative capacity. Taken together, these findings define an emerging role for the microbiota within cecal crypts to maintain epithelial cell homeostasis in a manner that may enhance recovery in response to the physiological stress imposed by the process of surgery.

**NEW & NOTEWORTHY** This study provides novel insight into the process by which surgical injury places the intestinal epithelium at risk for colonization by pathogenic microbes and impairment of its regenerative capacity via loss of its microbiota. We show that fecal transplant restores crypt homeostasis in association with repopulation of the microbiota within cecal crypts.

## Abstract (precis)

This study provides novel insight into the process by which surgical injury places the intestinal epithelium at risk for colonization by pathogenic microbes and impairment of its regenerative capacity via loss of its microbiota. We show that fecal transplant restores crypt homeostasis in association with repopulation of the microbiota within cecal crypts.

the intestinal microbiota are physically distanced from the intestinal epithelium in both the small intestine and colon in healthy hosts (13, 29). In the small intestine, Paneth cells synthesize and secrete antimicrobial peptides and proteins to distance and ostensibly protect against microbial invasion (8, 29). In much of the large intestine, there is a thick mucus bilayer that overlies epithelial cells in which the inner layer is firmly attached to the underlying epithelium providing a physical separation of the colonic epithelium from the microbiota (14). The cecum, however, has unique properties in this regard whereby the microbiota are in close contact with the epithelial surface allowing for dense colonization of its crypts by commensal bacteria (23, 27). Yet the functional role of the microbiota within cecal crypts is unknown. One hypothesis that has been put forth is that cecal crypt microbiota act as reservoirs from which repopulation occurs following antibiotic treatment (27). Others propose that cecal crypts stabilize intestinal stem cells and support intestinal epithelia restitution (31). In support of this latter hypothesis is the observation that intestinal stem cells produce the microbial pattern recognition receptors nucleotide-binding oligomerization domain-containing protein 2 (NOD2) and Toll-like receptor 4 (TLR4) (10, 19, 31) and that bacterial cell wall products (i.e., muramyl dipeptide) can activate NOD2 in leucine-rich repeat-containing G protein-coupled receptor 5 (Lgr5)-positive stem cells and promote stem cell survival (20). Thus there is likely an important interaction between the microbiota at its interface with the crypt epithelium that promotes cellular homeostasis and potentially recovery from systemic stress such as surgical injury.

Surgery is a modern intervention that involves the imposition of a finite period of starvation, antibiotic exposure, and major tissue injury and stress. While recovery from major surgery is the norm, most complications that develop are due to pathogens that use the gut as their primary site of colonization (15). What effect, if any, surgery has on the microbiota-epithelial interface and how such changes might affect the ability of the intestinal epithelia to regenerate remain undefined. Here we sought to understand the effect of the process of surgery on the microbiota within the confines of cecal crypts owing to their close proximity to stem cells. Stem cells self-renew to maintain their numbers and differentiate into specialized epithelial cells along the crypt-villus axis in the adult intestine, a function that becomes especially important after injury (30). We used a well-established model of lethal gut-derived sepsis developed in our laboratory (1) and demonstrated that the process of surgery including preoperative starvation (S), preoperative antibiotic treatment (A), and a 30% hepatectomy (H) leads to depletion of microbiota within cecal crypt in association with altered crypt homeostasis as judged by decreased epithelial regeneration. Introduction of pathogens into the cecal compartment when crypts are depleted of their microbiota permitted pathogen occupation and lethal sepsis to develop. Introduction of a fecal microbiota transplant (FMT) via enema in this model populated the cecal crypts with microbiota, prevented pathogen occupation, and maintained epithelial cell homeostasis as judged by stem cell proliferative capacity. Taken together, these findings define a role and spatial context in which the commensal microbiota within cecal crypts are involved in epithelial homeostasis.

## MATERIALS AND METHODS

### 

#### Mouse model of lethal gut-derived sepsis.

**Fig. 1.**
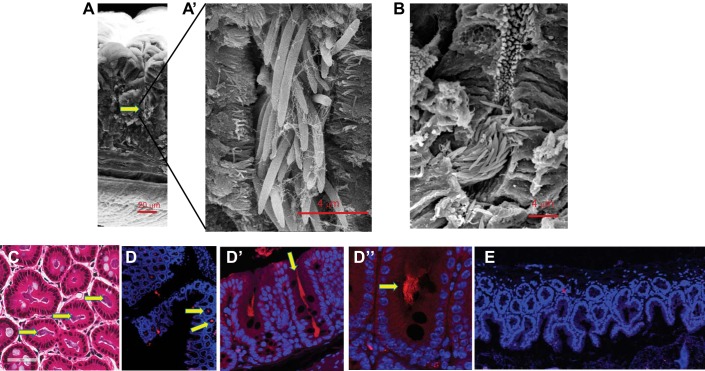
Colonization of cecal crypts by commensal microflora. *A*–*D*″: commensal microflora in cecal crypts of untreated mice analyzed by scanning electron microscopy (SEM; *A* and *B*), hematoxylin and eosin staining (*C*), and fluorescence in situ hybridization (FISH) with universal bacterial probe labeled with Texas red (*D* and *D′*). *E*: nonsense Texas red probe to confirm FISH specificity. Yellow arrows depict microbiota within cecal crypts.

All experiments were approved by the Institutional Animal Care and Use Committee, and Institutional Biosafety Committee protocols at the University of Chicago (IACUC Protocol 71744, IBC Protocol 968). Seven-to-nine-week-old male C57B/L6 mice weighing 18–22 g were used for all experiments. Mice were routinely fed tap water and Harland Teklad feed (Madison, WI) under 12-h light/dark cycles and were allowed to acclimate for at least 48 h before surgery. To mimic preoperative surgical conditions, mice were exposed to prophylactic antibiotics and a short period of starvation (H_2_O only). Approximately 16 h before surgery, mice were moved to wire floor cages to prevent coprophagy. Food was removed, and mice were allowed only tap water ad libitum. Each mouse received an intramuscular cefoxitin injection 30 min before surgery at a concentration of 25 mg/kg into the left thigh. Mice were then subjected to a midline laparotomy, 30% hepatectomy of the left lateral lobe of the liver via cautery, and a direct cecal inoculation of a pathogen community (PC; 200 µl of PC suspension injected via needle puncture into the cecum). Following the procedure, the midline incision was closed with suture and mice were returned to their cages. As we have previously described, this model results in gross signs of sepsis and mortality rates between 60 and 80% at 48 h (34). Mice that are not inoculated with the PC do not develop sepsis and recover to normalcy without any mortality. To determine the influence of the microbiota on cecal crypt homeostasis following surgical injury, reiterative experiments were performed whereby the microbiota were replenished 1 day after surgery using a FMT delivered by enema. The FMT was freshly prepared by harvesting the cecal contents of untreated healthy mice. Cecal contents were then suspended in sterile 10% glycerol at a concentration of 50 mg/ml. On *postoperative days 1* and *2* (POD1 and POD2), mice were given 1 ml of FMT suspension via enema using 10-Fr pediatric red rubber catheter. Chow was resumed on POD2 where mice ate ad libitum. The experimental protocol and treatment assignment is summarized in Fig. 1.

#### A human PC as an infectious inoculum.

We previously characterized a PC isolated from the stool of a patient with severe sepsis syndrome and prolonged critical illness (34, 35) that was collected under our Institutional Review Board protocol (IRB 16494B). The PC served as the infectious inoculum in our current experiments. The PC consists of four members: *Candida albicans*, tetracycline-resistant *Enterococcus faecalis*, multidrug-resistant *Klebsiella oxytoca*, and multidrug-resistant *Serratia marcescens*. The individual pathogens were plated on tryptic soy agar plates from individual frozen stocks and grown overnight at 37°C. Colonies were suspended in liquid tryptic soy broth (TSB) medium, subgrown for 1 h, and then adjusted to an OD600 of 0.2. All four microbe species were then combined together in equal volumes. The bacterial suspension was centrifuged at 6,000 rpm for 10 min, the excess TSB was removed, and the remaining pellet was resuspended in 10% glycerol. The resulting microbial suspension was used for all cecal injections as described below.

#### Cecal tissue fixation.

The cecum was cut into small pieces (~5 mm) and fixed in freshly prepared 4% paraformaldehyde in phosphate-buffered saline for 24 h at 4°C. Tissues were then washed with 70% ethanol, embedded in paraffin blocks, and sectioned at 5 µm. Paraffin-embedded sections were deparaffinized by baking at 60°C during 3 h, incubated in xylene twice for 5 min, and then incubated in 100% ethanol, twice, for 1 min.

#### Bacterial staining using fluorescence in situ hybridization.

For visualization of microbial organisms, we used probes designed by Miacom Diagnostics, which uses a next generation fluorescence in situ hybridization (FISH) beacon-based technology providing more specific binding properties over classic FISH methods. A bacterial universal probe to visualize microbiota was labeled by Texas red. The specificity of the probe was verified using a nonsense Texas red probe that did not show any red staining inside the crypts (Fig. 1*E*).

To visualize the PC species, microbe-specific probes were labeled with different spectrum fluorescence ranges for use in a FISH multiplex assay: *E. faecalis* Alexa Fluor 647 (purple), *S. marcescens* Alexa Fluor488 (green), *K. oxytoca* Alexa Fluor 555 (red), and *C. albicans* ATTO 425 (blue). Assays were performed according to the protocol provided by company. Confocal microscopy was performed on a Leica SP5 II AOBS tandem scanner spectral confocal system on a DMI6000 microscope and controlled by LASAF software (version 2.8.3). Tiled capture of Z stacks was automatically collected and images were merged using the stage scanning feature of the microscope and LASAF software. Four channels were collected at each location using sequential excitation (excitation: 405, 488, 561, and 633; emission: 412–452, 495–537, 578–631, and 654–755 nm pass bands) on either photomultiplier or HyD hybrid detectors. Objectives used were ×20, NA 0.7 dry, ×63, NA 1.40 oil, and ×100, NA 1.45 oil (Leica). The amount of crypts populated by normal microbiota (universal Texas red probe) vs. the PC (multiplex FISH) was determined by blinded observer.

#### Hematoxylin and eosin analysis.

Analysis of hematoxylin and eosin (H&E)-stained slides (5 mice/group, groups nontreated, SA, SAH, SAHPC, and SAHPC + FMT was performed by a pathologist blinded to a treatment. Image analysis was based on the presence of apoptosis (surface and crypt) and polymorphonuclear cells (surface, crypt, and serosa). The average counts performed in 10 (or less, available) high-power fields (×400) were compared in the five groups. The depth of the crypts was assessed by measuring the distance from the base of the crypt to the flat margin of cecal mucosa.

#### LGR5 staining.

To determine the effect of the microbiota on epithelial stem cells, we examined cecal crypts for the expression of Lgr5 using a RNAscope 2.5 HD Detection Reagent (brown) (Advanced Cell Diagnostics, Hayward, CA) (32). Assays were performed according to the protocol provided by the company. Three-dimensional (3D) quantitative analysis of stained tissues was performed using a high resolution (×40, NA 0.95) digitized image collected on the Pannoramic Scan (3D HISTECH; Perkin Elmer). The percentage of Lgr5-positive cells in crypts and the mean value of Lgr5-positive cells distributed along the length of the crypts were calculated using the ImageJ ImmunoRatio plugin (28) with constant thresholds.

#### Ki67 staining.

Formalin-fixed paraffin-embedded sections of 5-µm thickness were preheated at 60°C for 30 min, deparaffinized in xylene (2× 5 min), rehydrated in graded ethanol (100%, 3 min; 95%, 3 min, and 70%, 3 min) and washed in PBS for 5 min. Antigen retrieval was performed in Tris-EDTA-Tween 20 buffer (10 mM Tris base, 1 mM EDTA, and 0.05% Tween 20), pH 8.5, for 20 min in the steamer at 99°C, cooled to room temperature (RT), and washed in PBS. Sections were circled with a hydrophobic barrier pen and rinsed with TBS + 0.1% Tween 20 solution. Blocking was performed with TBS containing 0.1% Tween 20 + 5% BSA for 30 min at room temperature. Incubation with *K*_i_-67 Specific Rabbit Monoclonal SP6 Antibody (cat. no. RM-9106; ThermoScientific) diluted at 1:200 in TBS containing 0.1% Tween 20 + 2% BSA was performed overnight at 4°C followed by washing in TBS + 0.1% Tween 20. Goat anti-rabbit IgG (cat. no. A-21428, Alexa Fluor 555; Invitrogen) diluted at 1:500 in TBS containing 0.1% Tween 20 + 2% BSA were then applied for 1 h at RT. After being rinsed with TBS + 0.1% Tween 20, the ProLong Diamond Antifade Mountant with DAPI (Molecular Probes) was used for mounting. Confocal microscopy was performed on a Leica SP5 II tandem scanner spectral confocal system. Relative depth of proliferative zone was determined as a ratio of the depth of proliferative zone to the depth of crypt.

#### Terminal deoxynucleotidyl transferase dUTP-mediated nick-end labeling staining.

Terminal deoxynucleotidyl transferase dUTP-mediated nick-end labeling (TUNEL) staining was performed using ApopTag Plus Peroxidase In Situ Apoptosis Detection Kit (Millipore) according to the manufacturer’s protocol. A 3D quantitative analysis was performed with the 3D Histech Pannoramic Scan150 (×40). The percentage of TUNEL-positive cells in crypts was calculated by the custom ImageJ macro. Fully imaged crypts were highlighted using an automated DAB-positive cell segmentation, and their location and distance from crypt mouth to base were calculated as a percentage of the total.

#### Scanning electron microscopy.

Cecal tissues were fixed in 3% glutaraldehyde buffered with 0.1 M phosphate buffer, pH 7.2, washed with 0.1 M phosphate buffer, and dehydrated in a graded ethanol solution in water (30% increased gradually to 100%; 20 min each). The samples were dried with a Leica CPD300 critical point dryer and coated with Pt(80)/Pd (20) of an 8- to 12-nm thickness by using a Cressington sputter coater (model 208HR). Samples were viewed with a FEI Nova NanoSEM230 scanning electron microscope (SEM).

#### Isolation of crypts and formation of organoids.

To determine the regenerative capacity of the cecal epithelium in mice across the various treatment groups, we determined the ability of harvested cecal epithelia to form into mature 3D organoid structures ex vivo. Mice were euthanized accordingly to the standard euthanasia protocol of the University of Chicago Animal Resource Center. Whole ceca were extracted and transferred into petri dishes with ice-cold d-PBS (GIBCO). The surrounding fat and mesentery were carefully removed and a longitudinal incision was made along the length of the cecum. Cecal contents were removed with gentle swirling and cecal tissues were cut into 2-mm strips using razor blades. Tissue pieces were then gently cleaned of remaining cecal contents by gentle pipetting in d-PBS. Tissue pieces were incubated in 5 mM EDTA in d-PBS for 30 min at 5°C to loosen the crypts from surrounding tissue. Tissues were then washed in 5 ml of Advanced DMEM/F-12 (ADF; Advanced Dulbecco’s Modified Eagle’s Medium/Ham’s F-12; GIBCO) with gentle shearing using a 10-ml pipette. This was repeated three times and loose crypts were collected with each step. Resulting suspension was filtered through a 100-μm filter and centrifuged at 300 *g* for 5 min. The pellet was then resuspended in 15 ml of ADF and centrifuged at 150 *g* for 2 min. The pellet was resuspended in 3 ml of ADF and crypts were counted using AmScope and adjusted to 150 crypts per 50 μl of solution. The suspension was centrifuged again at 150 *g* for 2 min and the pellet was resuspended in a corresponding volume of Matrigel (Corning). Crypts in Matrigel were then again counted and recorded. 12-well plates were warmed up at 37°C for 30 min before use. Fifty microliters of Matrigel containing cecal crypts were placed at the center of each well and allowed to set for 15 min at 37°C and 5% CO_2_. Organoid media warmed to 37°C in a water bath were added to each well (200 µl/well). After 3 days of incubation, media were replaced with the fresh one. After 5 days, organoids were counted under light microscopy and the percent organoid yield was calculated and normalized to the initial crypt numbers in Matrigel.

#### Organoid medium preparation.

The organoid medium was made up of 50% primary culture media (Advanced DMEM/F-12, 100 units/ml penicillin/streptomycin, 2 mM l-glutamine, and 20% FBS) and 50% conditioned media from L-WRN supportive cell line obtained as a gift from Dr. Tong Chuan He (Department of Surgery, University of Chicago). The L-WRN conditioned media were rich in Wnt3a, R-spondin, and Noggin, factors necessary for formation and maintenance of cecal organoids in culture. This culture media were stored at −80°C and used within 6 mo without any observable loss of action. A 50-ml aliquot was thawed immediately before use and supplemented with additional 50 ng/ml mouse recombinant EGF, 1× B-27, 1× N2, 500 ng/ml R-spondin, 10 µM Y27623, and 1 µM Jagged1.

#### Statistical analysis.

Sampling details are given in figure legends. Statistical analysis was performed using SigmaPlot and GraphPad Prism softwares. Student’s *t*-tests were used with a significance determined as a *P* < 0.05. Nonparametric statistical analysis with Bonferroni correction for multiple comparisons was used for crypts’ depth analysis.

## RESULTS

### 

#### Cecal crypts are tightly packed at their base with microbiota.

Cecal and proximal colon crypts are considered to be a unique niche colonized by the commensal microflora (23). We performed SEM to obtain high-resolution images of microbes inside the crypts and found that microbes are organized into compact bundles at the base of crypts (Fig. 1, *A* and *B*), predominantly representing by long rods (~4 µm) (Fig. 1, *A′* and *B′*). H&E staining (Fig. 1*C*) and FISH using a Texas red universal bacterial probe (Fig. 1, *D* and *D*″) demonstrated that most of the crypts were occupied by bacteria colonizing the full length of crypts (Fig. 1D*′*) including the base of crypts (Fig. 1*D*″). FISH specificity was verified using a nonsense Texas red probe (Fig. 1*E*).

#### Major surgery results in a dramatic loss of microbiota from cecal crypts.

**Fig. 2.**
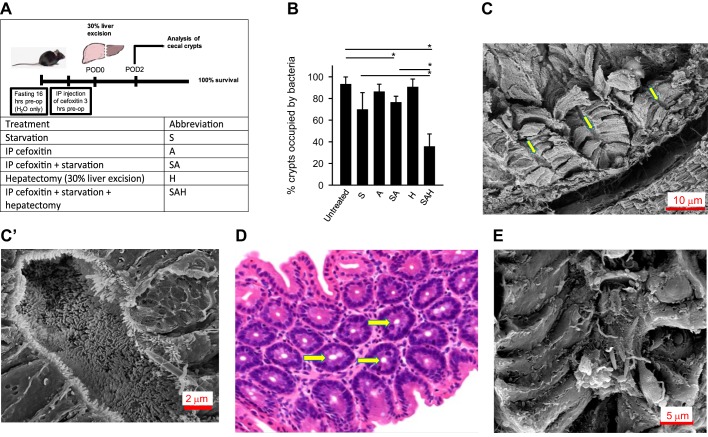
Loss of microbiota in cecal crypts of mice subjected to process of surgery. *A*: experimental design to test the effect of individual treatments on crypt microbiota colonization. *B*: quantitative analysis of %crypts occupied by microbiota via hematoxylin and eosin (H&E) staining; *n* = 5 mice/group, 3 cecal sections per mouse, 4,000 crypts examined/group. *P < 0.01. C and C′: SEM analysis of cecal crypts in SAH group demonstrating empty crypts. *D*: H&E staining of cecal tissues in SAH group. Yellow arrows depict empty crypts. *E*: SEM analysis of cecal crypts in SAH group demonstrating crypts sparsely colonized by bacterial cells. S, preoperative starvation; A, preoperative antibiotic treatment; H, 30% hepatectomy.

Routinely, patients undergoing elective surgery undergo an overnight fast to avoid aspiration of food contents when general anesthesia and endotracheal intubation are performed. In addition, it is routine to administer a prophylactic dose of parenteral antibiotics, which is usually a second generation cephalosporin (i.e., cefoxitin). To mimic this practice in our mouse model (Fig. 2*A*), we subjected mice to a 16-h fast before surgery with unlimited access to drinking water. An intramuscular injection of cefoxitin was administered 1 h before surgery. The surgery involved a partial liver resection (30% hepatectomy), an otherwise completely recoverable procedure in mice. Normal feeding was restored on POD2 (48 h postoperatively). All mice recovered from surgery without any morbidity during the length of follow up. A subset of mice was euthanized on POD2, and cecal tissues were fixed and cecal crypts analyzed. SEM, H&E staining, and FISH staining using universal Texas red probe were used for the analysis. Quantitative analysis was performed based on the H&E staining. Results demonstrated that starvation alone (S) or in combination with preoperative antibiotics (A) (i.e., S + A or SA) treatment resulted in a small increase in the number of crypts evacuated of bacteria; however, neither antibiotic treatment alone (A) nor hepatectomy alone (H) had a significant effect on crypt colonization by microbes (Fig. 2*B*). When SA mice were subjected to hepatectomy (SAH group), the level of crypt evacuation of bacteria was significantly increased from (25 ± 5% evacuation in SA) to (70 ± 8% evacuation in SAH). The majority of SAH crypts were completely devoid of microbiota (Fig. 2, *C* and *D*), and the remaining crypts were sparsely colonized by bacterial cells (Fig. 2*E*). These results demonstrate that the process of major surgical intervention including starvation-positive and antibiotic-positive hepatectomy (SAH) results in a profound loss of the commensal microbiota from cecal crypts. The SAH group was used for further experiments.

#### Crypt evacuation of bacteria is associated with abnormal Lgr5 activation and localization, an increase in the number of TUNEL-positive cells, and reduced epithelial regenerative capacity.

**Fig. 3.**
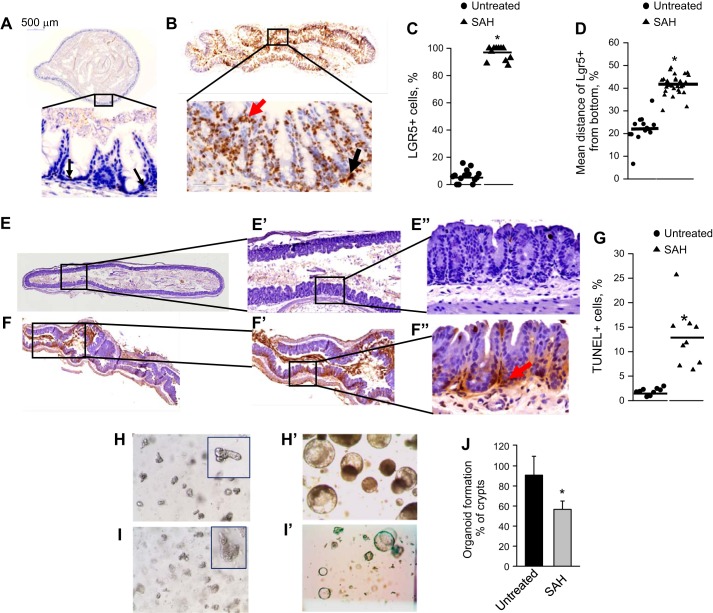
Crypts evacuated of microbiota are characterized by distorted homeostasis. *A*–*D*: in situ hybridization to detect leucine-rich repeat-containing G protein-coupled receptor 5 (Lgr5)-positive cells in nontreated (*A*) and SAH-POD2 (*B*) groups. Black arrow depicts Lgr5-positive cells at the bottom, and red arrow depicts Lgr5-positive cells at the top of crypts. *C*: relative intensity of Lgr5-positive cells in crypts. *D*: mean distance of Lgr5-positive cells from the bottom of crypts; *n* = 3 mice, 4 segments of each cecum = 12 samples/group. *P < 0.01. *E*–*G*: erminal deoxynucleotidyl transferase dUTP-mediated nick-end labeling (TUNEL) staining of nontreated (*E* and *E″*) and SAH-POD2 groups (*F* and *F″*). G: the %TUNEL-positive cells in crypts. *n* = 3 mice, 3 segments of each cecum = 9 samples/group. *P < 0.01. *H*–*J*: formation of organoids by crypts isolated from nontreated (*H* and *H″*) and SAH-POD2 (*I* and *I″*) groups. *H* and *I*: images of isolated crypts in Matrigel. *H′* and *I′*: images of formed organoids in 7 days after crypt seeding. *J*: %organoid formation normalized to total amount of seeded crypts; *n* = 3 mice/control group, 4 mice per postsurgery group, 4 Matrigel wells/per cecum, *n* = 12–16/group. *P < 0.01.

The close proximity of compact bundles of microbial cells to intestinal stem cells within the base of crypts raises the question as to what effect, if any, their elimination might have on stem cell function. To address this, we compared healthy untreated mice to mice treated by SAH. We have first analyzed cecal crypts for the presence of the LGR5, a biomarker of intestinal adult stem cells (5). In nontreated mice, in situ hybridization (ISH) with Lgr5 mRNA probe demonstrated low numbers of Lrg5-positive cells (2–4) localized at the bottom of crypts (Fig. 3*A*), which is in agreement with that reported by others (6). However, in SAH-treated mice, Lgr5 expression was significantly increased (Fig. 3, *B* and *C*), and Lgr5-positive cells were found to be abnormally distributed along the base-top axis and at the tips of the crypts (Fig. 3, *B* and *D*). TUNEL staining revealed extensive and significant regions in cecal tissues of TUNEL-positive cells in crypts (Fig. 3, *F* and *F″*) that was significantly higher compared with untreated controls (Fig. 3, *E*, *E″*, and *G*).

Finally, cecal crypts harvested from SAH-treated mice were significantly impaired in their capacity to form organoids (Fig. 3, *H* and *J*) suggesting that the LGR5-positive cells observed to be markedly increased were likely disabled as functional stem cells.

#### Pathogenic bacteria are able to occupy cecal crypts devoid of their microbiota.

**Fig. 4.**
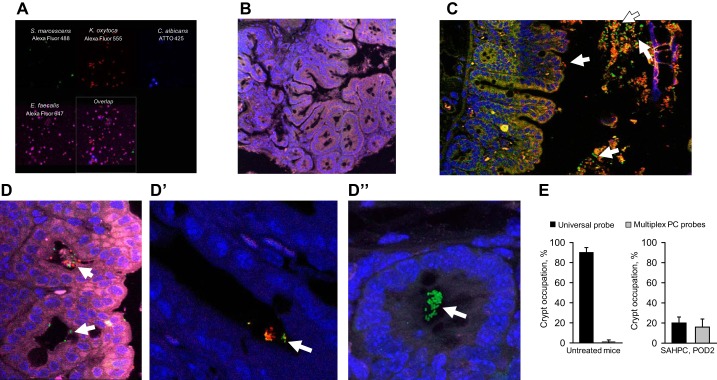
Pathogens from mice cecally inoculated with a 4-member pathogen community (PC) occupy empty cecal crypts. *A*: multiplex fluorescent in situ hybridization of *Klebsiella pneumoniae*, *Serratia marcescens*, *Enterococcus faecalis*, and *Candida albicans* inside the community using beacon-based custom probes (Miacom). *B*: multiplex PC FISH of cecal tissues in untreated mice demonstrating that PC probes do not hybridize to commensal microbiota. *C* and *D″*: multiplex PC FISH images of cecum SAHPC, POD2 mice demonstrating the presence of pathogenic bacteria in lumen (*C*) and crypts (*D* and *D″*). *S. marcescens*: green; *K. oxytoca*: red; and *E. faecalis*: purple fluorescence. *E*: comparative analysis of the occupation of crypts by commensal (universal probe) vs. pathogenic bacteria (multiplex PC probes) in untreated mice (*left*) and SAHPC, POD2 mice (*right*); *n* = 3 mice per group.

Previous work from our laboratory demonstrated that introduction of a four member human PC harvested from the stool of a critically ill patient into the cecum of SAH-treated mice resulted in a high mortality rate approximating 80% (34). The four-member PC consisted of *K. pneumoniae*, *S. marcescens*, *E. faecalis*, and *C. albicans* and was previously isolated from a critically ill patient’s stool sample (identified as ICU1-2) and characterized for its virulence phenotype and antibiotic resistance (34, 35). We hypothesized that the empty crypts observed in SAH-treated mice would be occupied by the PC when it was introduced into the cecum via direct puncture in SAH-treated mice (now referred to as SAHPC-treated mice). To test this, we developed a multiplex assay using FISH probes that identified each member of the PC (multiplex PC FISH) (Fig. 4*A*). As a negative control, we used cecal tissues from untreated mice. Negligible hybridization was found with the normal microbiota with a few positive signals corresponding to commensal *E. faecalis* (Fig. 4*B*). Multiplex PC FISH identified *S. marcescens*, *K. oxytoca*, and *E. faecalis* but not *C. albicans* in the lumen (Fig. 4*C*) and inside the crypts of the cecum of mice subjected to SAHPC (Fig. 4, *D* and *D″*). Comparative analysis of crypt occupation by commensal vs pathogenic bacteria revealed negligible signals in untreated mice where 90-100% of crypts were colonized by commensal microflora (Fig. 4*E*, *left*). However, in SAHPC-treated mice, only ~20% of the crypts were occupied by bacteria that were recognized by both the Universal bacterial probe and the multiplex PC FISH probe (Fig. 4*E*, *right*).

#### FMT leads to recolonization of cecal crypts by the commensal microbiota and epithelial homeostasis.

**Fig. 5.**
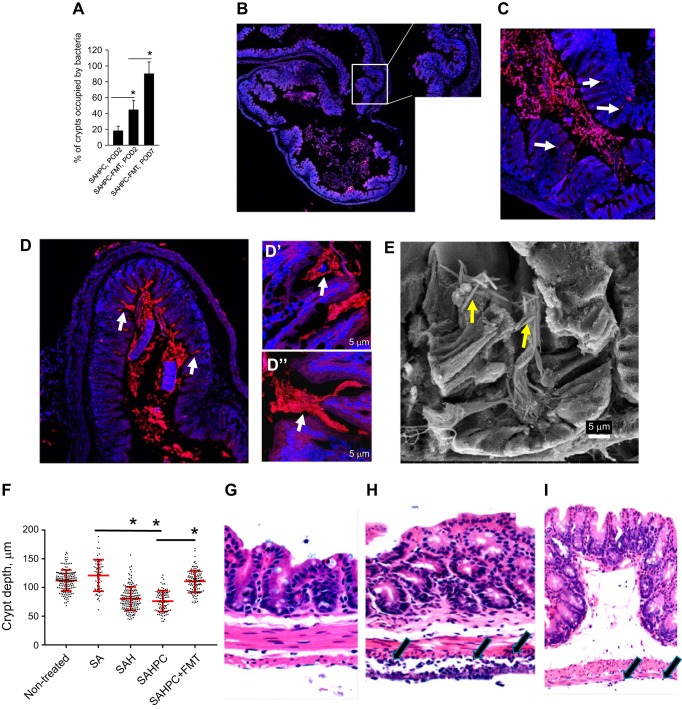
Fecal microbiota transplantation leads to repopulation of cecal crypts by commensal microbiota. *A*: quantitative analysis of crypts colonized by microbiota; *n* = 5 mice/group, 3 cecal sections per mouse, 4,000 crypts/group, *P < 0.05. *B*: multiplex PC FISH image of cecum SAHPC + FMT, POD3 demonstrating absence of pathogenic bacteria in cecal crypts. *C* and *D″*: universal bacterial FISH staining of SAHPC + FMT, POD3 (*C*) and SAHPC + FMT, POD7 (*D* and *D″*). Bacteria in crypts are depicted by white arrows. *E*: SEM analysis of cecal crypts in SAHPC + FMT, POD7 samples demonstrating repopulation of microbiota. Long rods bacteria are shown by yellow arrows. *F*: quantitative analysis of crypts′ depth; *n* = 5 mice/group, ~50–200 crypts/group, *P < 0.01 (nonparametric statistical analysis with Bonferroni correction for multiple comparisons). *G*–*I*: H&E staining demonstrating no serositis in SAH (*G*), severe serositis in SAHPC (*H*), and mild serositis in SAHPC + FMT (*I*) groups.

To verify the role of the microbiota to maintain crypt cell homeostasis, we administered a FMT to SAHPC-treated mice (this group is now termed SAHPC + FMT) on POD1 and again on POD2 via enema. Results indicated that the FMT led to an increase of cecal crypts occupation by commensal microbiota on POD2 when compared with SAHPC-treated mice that was further increased on POD7 (Fig. 5*A*). The multiplex PC FISH analysis at POD2 and POD3 demonstrated negligible amounts of pathogenic bacteria in the cecal lumen and crypts (Fig. 5*B*), whereas the universal bacteria FISH identified abundant colonization of lumen and crypts (Fig. 5, *C* and *D″*) suggesting that the FMT administration provided enough microbiota to outcompete the PC. SE analysis demonstrated the appearance of long rods bacteria in the crypts (Fig. 5*E*) similar to that seen in nontreated mice. Comparative histological analysis of groups as controls (nontreated), SA, SAH, SAHPC, and SAHPC + FMT mice was performed to compare crypt depth between groups. The analysis revealed a significant reduction of crypt depth in SAH and SAHPC groups that was reversed to normal by FMT treatment (Fig. 5*F*) suggesting that evacuation of the microbiota led a reduction in crypt depth which can be reversed by repopulation of crypts with microbiota. Histological analysis of polymorphonuclear cells (PMNs) revealed no differences between PMNs on the surface of the crypt epithelium across all groups. No PMNs were detected at the base crypts of nontreated mice. PMNs were present in the crypts of other groups but without statistical differences in between the groups. The most notable difference in the degree of PMN infiltration was observed in the serosa. There was no serositis (PMNs in serosa) in all groups without infection (nontreated, SA, and SAH) (Fig. 5*G*). However, severe serosal inflammation (i.e., serositis) was present in SAHPC group (Fig. 5*H*), which was markedly attenuated in the SAHPC + FMT group (Fig. 5*I*). Severe mucosal friability/loss of mucosa was present in SAHPC group. These data demonstrate that severe inflammation can be linked to occupation of crypts by the PC but not to crypt evacuation of their microbiota alone. The capacity of FMT to suppress inflammation may be due, in part, to its ability to successfully compete with the PC for crypt occupation.

**Fig. 6.**
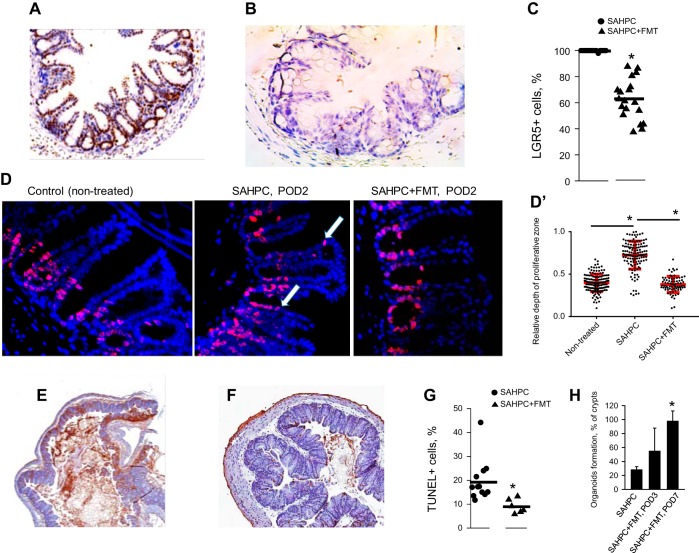
Fecal microbiota transplantation restores crypt cell homeostasis. *A*–*C*: in situ hybridization to detect Lgr5-positive cells in cecal crypts of SAHPC (*A*) and SAHPC + FMT (*B*). Quantitation of Lgr5-positive cells is displayed in *C*; *n* = 3 mice/group, 6 sections/mouse. *P < 0.01. *D* and *D′*: immunohistochemical staining for Ki67. White arrows depict localization of proliferating cells at the top of crypts of SAHPC, POD2 mice. The relative depth of proliferation was estimated as a ratio of proliferative depth to the crypt depth (*D′*); *n* = ~150 crypts/group, *P < 0.0001. *E*–*G*: TUNEL assay of cecal samples from SAHPC (*E*) and SAHPC + FMT-treated mice (*F*). Quantitation of TUNEL-positive cells in cecal crypts is displayed in *G*; *n* = 3 mice/group, 3 sections/mouse. *P < 0.01. *H*: %organoid formation from isolated crypts; *n* = 3 mice/group. *P < 0.001.

Repopulation of the crypts with microbiota in SAHPC + FMT mice was associated with normalization of Lgr5 expression (Fig. 6, *A*–*C*), which corresponded to normalized localization of proliferating cells to the stem cells compartment as judged by Ki67 immunohistochemical staining (Fig. 6, *D* and *D′*) and reduced apoptosis as judged by TUNEL staining (Fig. 6, *E* and *G*). The TUNEL staining data were confirmed by counting apoptotic cells per high-power field (HPF) on H&E-stained slides in a blinded fashion (5 mice/group). Crypt apoptosis was determined to be highest in SAH and SAHPC groups (mean 67/10 HPF and 30/10 HPF, respectively) as compared with SAHPC + FMT (mean 18/10 HPF) or SA (mean 8/10 HPF). The regenerative capacity of the cecal crypts, as judged by their ability to form organoids, was reestablished by FMT (Fig. 6*H*).

## DISCUSSION

The physiologic stress of major surgical intervention and its consequences on host recovery extend well beyond the surgical injury itself. The process of performing major surgery invariably involves a period of starvation and exposure to antibiotics which are known to have major effects on the microbiome (2, 22, 33). In addition, surgical injury itself may have an independent effect on the composition and function of the intestinal microbiome via mechanisms that remain unknown (11). Yet how the full process of surgery converges to alter the intestinal microbiota and what consequences, if any, this has on host resilience to infection remains unknown.

The unique structure and microbial content of the cecum, which are protected, in part, from the sweeping effect of the fecal stream, make it an ideal “window” in which to examine the effect of surgery on the intestinal microbiota. The cecum is a blind region of the intestine and is considered the first part of the large intestine. Similar to distal colon, the cecal epithelium does not have villi and its mucosa consists mainly of crypts (18). Paneth cells, which are abundant in the small intestine the function of which is to protect against enteric pathogens and control stem cells activity (8), are rare in cecum (18). As such, it is possible that the microbiota play an unappreciated role in regulating stem cell activity in the cecum. Others have shown that cecal microbiota have a dramatic effect on intestinal inflammation in mice following ileocecal resection (25, 26) confirming a more essential role for the cecal microbiota beyond the cecum.

**Fig. 7.**
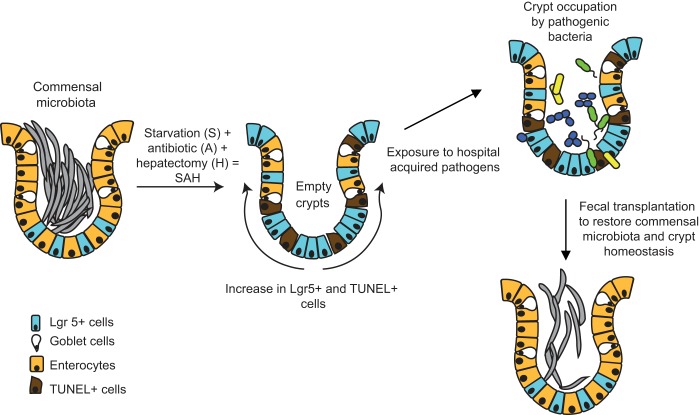
Role of commensal microbiota in crypt cell homeostasis. The process of surgery results in cecal crypts becoming evacuated of their microbiota, key drivers of crypt cell homeostasis as judged by Lgr5-positive cells and TUNEL staining. Occupation by pathogenic bacteria acquired during hospitalization can render crypts unable to drive the process of epithelial regeneration. Fecal microbiota transplantation may prevent or even rescue the process by which surgery and the exposure to pathogens leads to crypt dysfunction.

As summarized in Fig. 7, in the present study, we examined the effect of the process of surgery on the cecal crypt microbiota that may play a key and protective role in cellular and host homeostasis. We provide compelling evidence that cecal crypts’ microbiota can be destabilized during host physiological stress. Even a mild stress such as a limited period of starvation coupled with a single dose antibiotic treatment may lead to the partial crypt evacuation of their microbiota; however, the combination of starvation and antibiotic treatment with an otherwise recoverable surgical insult, such as a 30% hepatectomy, appeared to have a major impact on loss of the microbiota within cecal crypts. Taken together, these studies provide compelling evidence that loss of commensal microbiota at niches that affect stem cell function (i.e., crypts) may impact recovery. Clinical studies support the notion that the degree of starvation alone may adversely affect postoperative recovery (9, 17, 21, 24).

Crypt evacuation of microbiota was associated with abnormal localization of Lgr5-positive cells to the top of crypts, abnormal expansion of the proliferative zone toward the top of the crypts as visualized by Ki67 staining, and increased apoptosis, together suggesting a major disruption in crypt homeostasis. The consequences of crypt evacuation of its microbiota may be significant given the importance of stem cell regeneration on key epithelial function such as the intestinal barrier and its regenerative capacity. There are several potential mechanisms by which crypt microbiota may regulate stem cells. In ex vivo experiments with cecal organoids, others have demonstrated that the bacterial cell wall component muramyl-dipeptide stimulates the Nod2 receptor in Lgr5-positive stem cells and that triggers stem cell survival (20). Among other receptors that recognize bacterial components and are able to modulate stem cell function are TLR4 (19) and guanylyl cyclase C (3). Further studies are needed to elucidate these possible mechanisms.

Previous reports of Lgr5-positive cells localized to the upper crypts have only been descried for intestinal cancer (4, 16). Lgr5-positive cells normally do not localize to the top of crypts as they are restricted to base-level areas of the crypts (7). In the case of cancer, LGR5 expression is associated with increased cell proliferation and decreased apoptosis (12). In contrast, in the present study, increased apoptosis was observed and associated with LGR5-positive localization toward the top of the crypts when crypts were evacuated of their microbiota. This may suggest that when microbiota are eliminated from crypts and LGR5-positive cells abnormally locate to the top of crypts, host mechanisms dispose of them via apoptosis.

Importantly, in our experiments, the processes of LGR5 activation and apoptosis in cecal crypts of surgically stressed mice were reversible suggesting that major surgery induces a vulnerable but recovered state. Yet, colonization of crypts by pathogens imposes continuous disruption of crypt homeostasis. The importance of cecal crypt colonization by microbiota for normalization of homeostasis was confirmed by the introduction of FMT into intestine of SAHPC mice. After FMT, crypt become gradually filled with commensal microflora that is associated with normalization of localization Lgr5-positive, Ki67-positive cells, decrease TUNEL-positive cells, increased depth of crypts, and the restoration of the regenerative capacity of stem cells. Given that FMT was injected a day after the PC, it may prevent further colonization of crypts with pathogens or/and suppress the propagation of pathogens already present in crypts by successful competition of FMT microbiota with the PC. Finally, FMT may directly activate immune clearance of the pathogens.

The mechanistic details by which FMT drives the guts’ regenerative capacity remains to be clarified. Whether there are specific species among the commensal microbiota that are critical for the protective effect of FMT will require further study. Given that fasting before and after surgery is known to have a negative impact on recovery, how the microbiota in cecal crypts influence both gut integrity and regenerative capacity, within and beyond the cecum, may elucidate the mechanisms by which starvation adversely affects outcome following major surgery. As such, further work in this field is warranted.

## GRANTS

This work was funded by National Institute of General Medical Sciences Grant 5R01-GM-062344 (to J. C. Alverdy) and the Society for Surgery of the Alimentary Tract (SSAT) Mentored Research Award 2015 (to M. Krezalek).

## DISCLOSURES

No conflicts of interest, financial or otherwise, are declared by the authors.

## AUTHOR CONTRIBUTIONS

A.Z., M.K., S.H., J.R.D., and N.B. performed experiments; A.Z., M.K., J.R.D., V.P.B., Q.G., O.Z., and J.C.A. analyzed data; A.Z., M.K., S.H., J.R.D., N.S., V.P.B., Q.G., O.Z., and J.C.A. interpreted results of experiments; A.Z., N.S., and O.Z. prepared figures; A.Z., M.K., O.Z., and J.C.A. edited and revised manuscript; O.Z. drafted manuscript; O.Z. and J.C.A. approved final version of manuscript.
